# Use of machine learning to examine disparities in completion of substance use disorder treatment

**DOI:** 10.1371/journal.pone.0275054

**Published:** 2022-09-23

**Authors:** Aaron Baird, Yichen Cheng, Yusen Xia

**Affiliations:** 1 Institute of Health Administration, Robinson College of Business, Georgia State University, Atlanta, Georgia, United States of America; 2 Institute for Insight, Robinson College of Business, Georgia State University, Atlanta, Georgia, United States of America; Flinders University, AUSTRALIA

## Abstract

The objective of this work is to examine disparities in the completion of substance use disorder treatment in the U.S. Our data is from the Treatment Episode Dataset Discharge (TEDS-D) datasets from the U.S. Substance Abuse and Mental Health Services Administration (SAMHSA) for 2017–2019. We apply a two-stage virtual twins model (random forest + decision tree) where, in the first stage (random forest), we determine differences in treatment completion probability associated with race/ethnicity, income source, no co-occurrence of mental health disorders, gender (biological), no health insurance, veteran status, age, and primary substance (alcohol or opioid). In the second stage (decision tree), we identify subgroups associated with probability differences, where such subgroups are more or less likely to complete treatment. We find the subgroups most likely to complete substance use disorder treatment, when the subgroup represents more than 1% of the sample, are those with no mental health condition co-occurrence (4.8% more likely when discharged from an ambulatory outpatient treatment program, representing 62% of the sample; and 10% more likely for one of the more specifically defined subgroups representing 10% of the sample), an income source of job-related wages/salary (4.3% more likely when not having used in the 30 days primary to discharge and when primary substance is not alcohol only, representing 28% of the sample), and white non-Hispanics (2.7% more likely when discharged from residential long-term treatment, representing 9% of the sample). Important implications are that: 1) those without a co-occurring mental health condition are the most likely to complete treatment, 2) those with job related wages or income are more likely to complete treatment, and 3) racial/ethnicity disparities persist in favor of white non-Hispanic individuals seeking to complete treatment. Thus, additional resources may be needed to combat such disparities.

## Introduction

According to the 2020 National Survey on Drug Use and Health (NSDUH), 58.7% (or 162.5 million people) were current users of tobacco, alcohol, or an illicit drug [1]. A total of 14.5 percent (or 40.3 million people) were found to have a substance use disorder [1]. 1.4 percent (or 4.0 million people) aged 12 or older in the U.S. “received any substance use disorder treatment in the past year, and 1.0 percent (or 2.7 million people) received substance use disorder treatment at a specialty facility in the past year” [1]. Further, it is well known that treatment can effectively reduce substance dependence and improve related factors, such as associated mental health conditions, criminal behavior, and access to employment [2, 3].

Unfortunately, though, health care is subject to disparities [4] and, specific to our study, substance use disorder treatment outcomes can vary between subgroups [1, 5–11]. Work in this area has found that African Americans often wait longer to receive substance use disorder treatment service for opioids than their white counterparts [12, 13], and are less likely to complete treatment [14, 15]. It has also been found that racial disparities can differ by substance used, such as for methamphetamines vs. alcohol [5, 16]. Further, the most recent National Healthcare Quality and Disparities Report (2019) noted that income and being uninsured, in addition to race, were significant underlying factors in the presence of disparities in health quality [17]. Other studies have found that health care quality disparities persist by gender and age [18] and for those with mental health disease [19, 20]. More generally, disparities including income and race have been shown to be associated with disparities in health care quality and outcomes, but were also shown to be improving (i.e., less disparities) between 2006 and 2012 [4] and persisting in some areas but decreasing in others more recently [17]. Thus, we have an opportunity to comprehensively assess disparities in substance use disorder treatment completion, inclusive of a variety of determinants as well as to examine how present findings compare to prior findings. We also have an opportunity to apply state-of-the-art methods.

One methodological approach with a lot of promise is the use of machine learning (ML). ML has been receiving a lot of attention lately in the context of health care [21, 22]. ML has been applied specifically to analysis of substance use disorder treatment, resulting in interesting findings [23] with improved granularity and accuracy in many cases [9]. For instance, using two-stage virtual twins method (random forest + logistic regression), racial disparities were found to be present in wait times for treatment of opioid users [12]. Using a series of XGBoost models, one study found a number of complex interactions in factors associated with treatment completion, such as longer treatment times typically improving chances of treatment success yet success probability attenuating somewhat when frequency of substance use was at the 75^th^ percentile or higher [24]. Finally, using data on Medicare beneficiaries with emergency department admissions for opioid overdoses, ML has been used to develop accurate opioid overdose prediction models [25].

While excellent research has been conducted in this area, we claim: 1) the types of disparities considered should be inclusive of not only race and ethnicity, but also other determinants, 2) application of ML-based methods may help to more accurately identify subgroups more likely to complete treatment, and 3) the use of counterfactual research designs can help to establish causality. Given these claims, the objective of this study is to determine which subgroups are the mostly likely in the U.S. to complete substance use disorder treatment, using a method that combines the strengths of ML with the strengths of counterfactual research designs.

Conceptually, our work builds upon a growing body of social determinants of health [26] and disparities research [27–29] seeking to understand how systematic differences in subgroups, identified by *intersections* of characteristics [30], result in health outcome variations. We specifically consider how patient demographics, substance use characteristics, and treatment characteristics, impact treatment completion. This approach is consistent with work seeking to understand where heterogenous treatment effects are present, especially in observational data [31–33]. Conceptually, we assume heterogeneity in health care processes and outcomes, where some subgroups experience more favorable outcomes than others. We also assume, however, that heterogenous treatment effects with respect to disparities are either not immediately obvious to those providing care or are not always evaluated in depth. We use the most recently available national data (2017–2019), without restricting by substance type [12], region [14, 24], or only focusing on racially-based disparities [15]. Our findings contribute to research by leveraging a causal ML approach, applied to a number of disparities, and ultimately elucidating where disparities are persisting. Our findings contribute to practice by helping those who provide care to identify and be cognizant of what types of treatment episodes are more likely to result in completed treatment, even if not immediately obvious to care givers.

## Data and methods

### Study design

This study utilizes a counterfactual research design toward identification of subgroup differences, specifically designed to blend causal inference and ML methods [12, 31, 34]. This type of analysis allows for identification of subgroups with heterogenous treatment effects as well as identification of factors causing such differences [12]. This study was approved as exempt by an IRB as the data is anonymized and publicly available.

### Data source and sample

The data for this study comes from the publicly available, nationally representative TEDS-D datasets from SAMHSA for years 2017–2019. SAMHSA provides aggregated data for both admissions and discharges from substance use disorder treatment programs, for participating states (e.g., in 2019, all U.S. states participated except for Oregon, Washington, and West Virginia) and the District of Columbia and Puerto Rico. We selected the discharge data, as opposed to the admission data, to assess effects on treatment completion. Each observation is for one discharge, rather than one individual, which means that one individual may have multiple observations in the data. For the years of data we analyzed, a total of 649,479 discharges were included in our analyses. This sample represents 13.3% of the data. Missing data is explained in the S1 Appendix.

### Measures

#### Dependent variable (First stage)

Reason for discharge (REASON) from a substance use disorder treatment program was chosen as the primary dependent variable for the first stage estimation. This variable is categorical with seven categories: 1) treatment completed, which was coded as ‘1’ in our analysis for successful completion of treatment, and 2) six other categories coded as ‘0’ in our analysis to capture unsuccessful completion (i.e., dropped out of treatment, terminated by facility, transferred to another treatment program or facility, incarcerated, death, and other that captures a life circumstance change, such as hospitalization or change of residence).

#### Disparity variables

**Fig 1:**
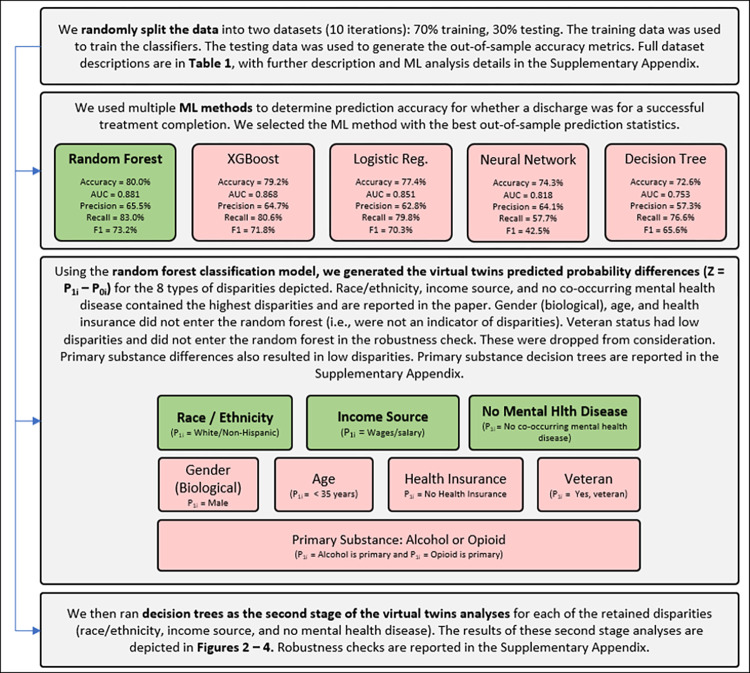
Research process flow chart.

While prior studies have typically focused on one type of disparity at a time, such as disparities related to race and ethnicity [12, 14, 15], we evaluate multiple disparities. As depicted in Fig 1, we evaluate disparities relative to: race/ethnicity (white non-Hispanic vs. rest), income source (incomes from wages or salary vs. rest), no mental health disease co-occurrence (no mental health disease co-occurrence vs. discharges for patients with mental health disease co-occurrence), gender (biological) (male vs. female), no health insurance (no health insurance vs. having health insurance), veteran status (yes a veteran vs. not a veteran), age (<35 years old vs. > = 35 years old), and primary substance (alcohol vs. rest; opioid vs. rest). Only some of these results are reported in this paper, with the rest included in the S1 Appendix, as some did not result in disparities being found or only at very low levels.

#### Explanatory variables

Three types of variables were included as explanatory variables. Some variables available in the TEDS-D data were dropped due to collinearity. See the S1 Appendix for details. Explanatory variables that were retained include:

- *Patient demographics*: Age at admission, gender (biological), race, ethnicity, marital status, education level, employment status at admission/discharge, veteran status, living arrangement at admission/discharge, primary income source, and arrests in the past month prior to the discharge.
- *Substance use characteristics*: Primary/secondary substance use, frequency of use at admission/discharge, and primary substance type reported at admission.
- *Treatment characteristics*: Type of treatment/service setting at admission/discharge, length of stay in treatment, referral source, detailed criminal justice referral, and previous substance use treatment episodes.

### Descriptive analyses

We generated descriptive statistics for the entire sample as well as for those who successfully completed treatment vs. those who did not. Additional descriptions, such as for missing data, was also generated and is provided in the S1 Appendix.

### Disparities analyses

In disparities research [12], we cannot observe different effects for the same observation for immutable characteristics. In the virtual twins approach, in the first stage estimation, a probability is determined for an outcome for every observation, which is treatment success (completion) in this study [34]. To estimate this probability, we follow prior work in this area [12, 24] and apply a machine learning approach. Specifically, we estimated probability of successful treatment completion with a random forest, XGBoost, a neural network, and a logistic regression. We applied a 70% training and 30% testing random data split with 10 iterations through the procedures to address variation due to randomness. We used the R package “h2o” to implement all the methods. For the neural network, we set the number of hidden cells to be (64,64), where the first 64 is the number of neurons in the first hidden layer and the second 64 for the second hidden layer. For all the other methods, we used the default settings. Random forest had the highest accuracy, AUC, and F1 and was selected as the finalist for the first stage estimations. Random forest is an ensemble method based on multiple decision trees. The model takes input covariate values (*X_i_, T_i_*), where *T_i_* is the binary indicator variable (subgroup variable) for whether an observation received and treatment or not, and the output is *P*(*Y_i_* = 1), i.e, the probability of successful completion, for that set of covariate values. For discharge *i*, we denote the probability as *P*_1*i*_ if the discharge is in the treatment group, and *P*_0*i*_ if otherwise.

To establish a counterfactual or a “virtual twin,” a second probability is calculated for every observation with the subgroup variable switched to its opposite value. The difference of these two probabilities is then calculated per observation (e.g., P(white non-Hispanic)–P(not white non-Hispanic)). This procedure was repeated for every disparity type evaluated, reported earlier. That is, we create a new variable for each discharge, defined as the difference in the probability for assuming discharge *i* is from the treatment group vs control group: *Z_i_* = *P*_1*i*_−*P*_0*i*_.

This difference is the primary variable in the second stage. In the second stage, we apply a decision tree to determine which factors, i.e., the same independent variables in the first stage other than the disparity variable under consideration, cause the probability difference [34, 35].

## Results

### Data description

The full dataset is described in Table 1. Some highlights are that the highest Reason for Discharge in the full sample was “treatment completed” (33.8%). The next highest categories are “transferred to another treatment program” (29.2%) and “dropped out of treatment” (22.8%). In the treatment not completed subgroup, 34.5% of discharges are for “dropped out of treatment,” while 44.1% are for “transferred to another treatment program.” Most of the treatment discharges in the full sample were from ambulatory outpatient centers (15.2% for intensive outpatient and 47.0% for non-intensive outpatient). 57.0% of discharges were for lengths of stay for between 1 and 30 days (57.0%).

**Table 1:** TEDS-D sample description (2017–2019) including differences for substance use disorder treatment completed vs. not completed subgroups.

			Categorical Description	Full Sample		Trt. Completed		Not Completed	
Variable	Abbr.	Value		Freq	%	Freq	%	Freq	%
**Year of client’s discharge from treatment**	**DISYR**		2017	162,063	25	56,168	25.6	105,895	24.6
			2018	232,365	35.8	78,219	35.6	154,146	35.9
			2019	255,051	39.3	85,271	38.8	169,780	39.5
**Reason for discharge**	**REASON**	**a**	**Treatment completed**	**219,658**	**33.8**	**219,658**	**100**	**-**	**0**
		**b**	Dropped out of treatment	148,174	22.8	-	0	148,174	34.5
		**c**	Terminated by facility	49,505	7.6	-	0	49,505	11.5
		**d**	Transferred to another trt program	189,410	29.2	-	0	189,410	44.1
		**e**	Incarcerated	14,954	2.3	-	0	14,954	3.5
		**f**	Death	1,156	0.2	-	0	1,156	0.3
		**g**	Other	26,622	4.1	-	0	26,622	6.2
**Race**	**RACE**	**a**	Alaskan Native	4,457	0.7	2,286	1	2,171	0.5
		**b**	American Indian	12,915	2	6,727	3.1	6,188	1.4
		**c**	Asian or Pacific Islander	16	0	11	0	5	0
		**d**	Black or African American	114,081	17.6	41,583	18.9	72,498	16.9
		**e**	White	455,413	70.1	148,282	67.5	307,131	71.5
		**f**	Asian	3,402	0.5	1,201	0.5	2,201	0.5
		**g**	Other single race	44,029	6.8	14,982	6.8	29,047	6.8
		**h**	Two or more races	8,730	1.3	2,754	1.3	5,976	1.4
		**i**	Native Hawaiian or Other Pacific Islander	6,436	1	1,832	0.8	4,604	1.1
**Ethnicity**	**ETHNIC**	**a**	Puerto Rican	25,619	3.9	8,721	4	16,898	3.9
		**b**	Mexican	13,979	2.2	7,425	3.4	6,554	1.5
		**c**	Cuban or other specific Hispanic	19,484	3	8,476	3.9	11,008	2.6
		**d**	Not of Hispanic or Latino Origin	577,254	88.9	189,838	86.4	387,416	90.1
		**e**	Hispanic or Latino, origin not specified	13,143	2	5,198	2.4	7,945	1.8
**Gender (Biological)**	**GENDER**	**a**	Male	402,735	62	152,620	69.5	250,115	58.2
		**b**	Female	246,744	38	67,038	30.5	179,706	41.8
**Martial Status**	**MARSTAT**	**a**	Never married	424,619	65.4	143,833	65.5	280,786	65.3
		**b**	Now married	81,081	12.5	27,115	12.3	53,966	12.6
		**c**	Separated	44,916	6.9	13,944	6.3	30,972	7.2
		**d**	Divorced/widowed	98,863	15.2	34,766	15.8	64,097	14.9
**Education**	**EDUC**	**a**	< Grade 9	34,945	5.4	9,556	4.4	25,389	5.9
		**b**	Grades 9 to 11	146,245	22.5	45,546	20.7	100,699	23.4
		**c**	Grade 12 (or GED)	307,664	47.4	105,530	48	202,134	47
		**d**	1–3 years of post-secondary	127,879	19.7	46,699	21.3	81,180	18.9
		**e**	4+ years of post-secondary	32,746	5	12,327	5.6	20,419	4.8
**Veteran Status**	**VET**	**a**	Yes	17,100	2.6	7,122	3.2	9,978	2.3
		**b**	No	632,379	97.4	212,536	96.8	419,843	97.7
**Sources of income/support**	**PRIMINC**	**a**	Wages/salary	166,161	25.6	59,486	27.1	106,675	24.8
		**b**	Public assistance	53,294	8.2	16,356	7.4	36,938	8.6
		**c**	Retirement/pension, disability	38,081	5.9	11,389	5.2	26,692	6.2
		**d**	Other	117,726	18.1	35,012	15.9	82,714	19.2
		**e**	None	274,217	42.2	97,415	44.3	176,802	41.1
**Age at admission (binned)**	**AGE**	**a**	12–14 years	2,397	0.4	928	0.4	1,469	0.3
		**b**	15–17 years	14,019	2.2	4,531	2.1	9,488	2.2
		**c**	18–20 years	20,224	3.1	6,228	2.8	13,996	3.3
		**d**	21–24 years	61,586	9.5	19,009	8.7	42,577	9.9
		**e**	25–29 years	125,202	19.3	38,701	17.6	86,501	20.1
		**f**	30–34 years	119,503	18.4	37,462	17.1	82,041	19.1
		**g**	35–39 years	96,632	14.9	31,099	14.2	65,533	15.2
		**h**	40–44 years	61,928	9.5	21,130	9.6	40,798	9.5
		**i**	45–49 years	51,372	7.9	19,136	8.7	32,236	7.5
		**j**	50–54 years	47,235	7.3	19,466	8.9	27,769	6.5
		**k**	55–64 years	45,188	7	19,851	9	25,337	5.9
		**l**	65 years and older	4,193	0.6	2,117	1	2,076	0.5
**Employment status at admission**	**EMPLOY**	**a**	Full-time	106,412	16.4	40,672	18.5	65,740	15.3
		**b**	Part-time	43,414	6.7	13,965	6.4	29,449	6.9
		**c**	Unemployed	251,461	38.7	75,712	34.5	175,749	40.9
		**d**	Not in labor force	248,192	38.2	89,309	40.7	158,883	37
**Employment status at discharge**	**EMPLOY_D**	**a**	Full-time	125,296	19.3	53,599	24.4	71,697	16.7
		**b**	Part-time	48,646	7.5	16,026	7.3	32,620	7.6
		**c**	Unemployed	243,200	37.4	73,430	33.4	169,770	39.5
		**d**	Not in labor force	232,337	35.8	76,603	34.9	155,734	36.2
**Living arrangement at admission**	**LIVARAG_A**	**a**	Homeless	107,181	16.5	45,769	20.8	61,412	14.3
		**b**	Dependent living	119,742	18.4	40,640	18.5	79,102	18.4
		**c**	Independent living	422,556	65.1	133,249	60.7	289,307	67.3
**Living arrangement at discharge**	**LIVARAG_D**	**a**	Homeless	89,380	13.8	34,363	15.6	55,017	12.8
		**b**	Dependent living	135,898	20.9	45,809	20.9	90,089	21
		**c**	Independent living	424,201	65.3	139,486	63.5	284,715	66.2
**Arrests in past 30 days prior to admission**	**ARRESTS**	**a**	None	595,125	91.6	199,691	90.9	395,434	92
		**b**	Once	47,759	7.4	17,336	7.9	30,423	7.1
		**c**	Two or more times	6,595	1	2,631	1.2	3,964	0.9
**Arrests in past 30 days prior to discharge**	**ARRESTS_D**	**a**	None	601,854	92.7	207,276	94.4	394,578	91.8
		**b**	Once	37,010	5.7	8,062	3.7	28,948	6.7
		**c**	Two or more times	10,615	1.6	4,320	2	6,295	1.5
**Previous substance use treatment episodes**	**NOPRIOR**	**a**	No prior treatment episodes	200,009	30.8	56,747	25.8	143,262	33.3
		**b**	One or more prior treatment episodes	449,470	69.2	162,911	74.2	286,559	66.7
**Type of treatment/ service setting at admission**	**SERVICES**	**a**	Detox, 24 hour, hospital inpatient	6,897	1.1	2,552	1.2	4,345	1
		**b**	Detox, 24 hour, free-standing residential	48,287	7.4	35,591	16.2	12,696	3
		**c**	Rehab/residential, hospital (non-detox)	346	0.1	271	0.1	75	0
		**d**	Rehab/residential, short term (< = 30 days)	130,461	20.1	70,011	31.9	60,450	14.1
		**e**	Rehab/residential, long term (> 30 days)	56,828	8.7	20,986	9.6	35,842	8.3
		**f**	Ambulatory, intensive outpatient	98,844	15.2	19,137	8.7	79,707	18.5
		**g**	Ambulatory, non-intensive outpatient	305,483	47	69,483	31.6	236,000	54.9
		**h**	Ambulatory, detoxification	2,333	0.4	1,627	0.7	706	0.2
**Length of stay in treatment (binned days)**	**LOS**	**a**	between 1 and 30 days	370,434	57	113,840	51.8	256,594	59.7
		**b**	between 31 and 45 days	44,270	6.8	12,203	5.6	32,067	7.5
		**c**	between 46 and 60 days	32,631	5	8,208	3.7	24,423	5.7
		**d**	between 61 and 90 days	51,719	8	16,802	7.6	34,917	8.1
		**e**	between 91 and 120 days	38,404	5.9	15,511	7.1	22,893	5.3
		**f**	between 121 and 180 days	43,901	6.8	18,949	8.6	24,952	5.8
		**g**	between 181 and 365 days	49,703	7.7	25,287	11.5	24,416	5.7
		**h**	greater than 365 days	18,417	2.8	8,858	4	9,559	2.2
**Referral Source**	**PSOURCE**	**a**	Individual (includes self-referral)	227,636	35	73,120	33.3	154,516	35.9
		**b**	Alcohol/drug use care provider	70,792	10.9	30,549	13.9	40,243	9.4
		**c**	Other health care provider	43,999	6.8	16,781	7.6	27,218	6.3
		**d**	School (educational)	1,865	0.3	655	0.3	1,210	0.3
		**e**	Employer/EAP	2,981	0.5	1,637	0.7	1,344	0.3
		**f**	Other community referral	91,898	14.1	23,195	10.6	68,703	16
		**g**	Court/criminal justice referral/DUI/DWI	210,308	32.4	73,721	33.6	136,587	31.8
**DSM diagnosis (SuDS 4 or SuCDS 19)**	**DSMCRIT**	**a**	Alcohol-induced disorder	2,068	0.3	1,130	0.5	938	0.2
		**b**	Substance-induced disorder	16,332	2.5	9,529	4.3	6,803	1.6
		**c**	Alcohol intoxication	19,504	3	18,108	8.2	1,396	0.3
		**d**	Alcohol dependence	103,287	15.9	41,766	19	61,521	14.3
		**e**	Opioid dependence	176,549	27.2	41,281	18.8	135,268	31.5
		**f**	Cocaine dependence	28,756	4.4	9,228	4.2	19,528	4.5
		**g**	Cannabis dependence	44,891	6.9	13,215	6	31,676	7.4
		**h**	Other substance dependence	96,169	14.8	26,190	11.9	69,979	16.3
		**i**	Alcohol use disorder	15,605	2.4	7,393	3.4	8,212	1.9
		**j**	Cannabis use disorder	12,688	2	4,920	2.2	7,768	1.8
		**k**	Other substance use disorder	10,242	1.6	3,191	1.5	7,051	1.6
		**l**	Opioid use disorder	7,772	1.2	2,449	1.1	5,323	1.2
		**m**	Cocaine use disorder	3,396	0.5	1,135	0.5	2,261	0.5
		**n**	Anxiety disorders	5,083	0.8	475	0.2	4,608	1.1
		**o**	Depressive disorders	6,861	1.1	678	0.3	6,183	1.4
		**p**	Schizophrenia/other psychotic disorders	1,923	0.3	188	0.1	1,735	0.4
		**q**	Bipolar disorders	2,824	0.4	286	0.1	2,538	0.6
		**r**	Attention deficit/disruptive beh. disorders	366	0.1	44	0	322	0.1
		**s**	Other mental health condition	95,163	14.7	38,452	17.5	56,711	13.2
**Substance use at admission (primary)**	**SUB1**	**a**	None	168,218	25.9	79,941	36.4	88,277	20.5
		**b**	Alcohol	49,874	7.7	17,947	8.2	31,927	7.4
		**c**	Cocaine/crack	76,767	11.8	23,627	10.8	53,140	12.4
		**d**	Marijuana/hashish	173,920	26.8	46,760	21.3	127,160	29.6
		**e**	Heroin	1,661	0.3	303	0.1	1,358	0.3
		**f**	Non-prescription methadone	56,429	8.7	15,220	6.9	41,209	9.6
		**g**	Other opiates and synthetics	2,260	0.3	724	0.3	1,536	0.4
		**h**	PCP	1,182	0.2	344	0.2	838	0.2
		**i**	Hallucinogens	96,848	14.9	28,112	12.8	68,736	16
		**j**	Methamphetamines/speed	6,070	0.9	1,618	0.7	4,452	1
		**k**	Other amphetamines	982	0.2	243	0.1	739	0.2
		**l**	Other stimulants	9,314	1.4	3,448	1.6	5,866	1.4
		**m**	Benzodiazepines	81	0	8	0	73	0
		**n**	Other tranquilizers	151	0	79	0	72	0
		**o**	Barbiturates	714	0.1	274	0.1	440	0.1
		**p**	Other sedatives or hypnotics	386	0.1	136	0.1	250	0.1
		**q**	Inhalants	317	0	90	0	227	0.1
		**r**	Over-the-counter medications	4,305	0.7	784	0.4	3,521	0.8
		**s**	Other drugs	-	0	-	0	-	0
**Substance use at admission (secondary)**	**SUB2**	**a**	None	3,060	0.5	644	0.3	2,416	0.6
		**b**	Alcohol	104,041	16	36,008	16.4	68,033	15.8
		**c**	Cocaine/crack	117,246	18.1	40,884	18.6	76,362	17.8
		**d**	Marijuana/hashish	169,697	26.1	59,614	27.1	110,083	25.6
		**e**	Heroin	40,725	6.3	13,632	6.2	27,093	6.3
		**f**	Non-prescription methadone	2,421	0.4	421	0.2	2,000	0.5
		**g**	Other opiates and synthetics	52,536	8.1	13,984	6.4	38,552	9
		**h**	PCP	1,967	0.3	754	0.3	1,213	0.3
		**i**	Hallucinogens	2,469	0.4	882	0.4	1,587	0.4
		**j**	Methamphetamines/speed	73,852	11.4	21,678	9.9	52,174	12.1
		**k**	Other amphetamines	7,387	1.1	2,283	1	5,104	1.2
		**l**	Other stimulants	3,453	0.5	926	0.4	2,527	0.6
		**m**	Benzodiazepines	35,957	5.5	11,521	5.2	24,436	5.7
		**n**	Other tranquilizers	170	0	35	0	135	0
		**o**	Barbiturates	504	0.1	145	0.1	359	0.1
		**p**	Other sedatives or hypnotics	2,051	0.3	738	0.3	1,313	0.3
		**q**	Inhalants	643	0.1	347	0.2	296	0.1
		**r**	Over-the-counter medications	549	0.1	190	0.1	359	0.1
		**s**	Other drugs	30,751	4.7	14,972	6.8	15,779	3.7
**Route of administration (primary)**	**ROUTE1**	**a**	Oral	218,128	33.6	94,109	42.8	124,019	28.9
		**b**	Smoking	162,850	25.1	52,494	23.9	110,356	25.7
		**c**	Inhalation	92,793	14.3	24,722	11.3	68,071	15.8
		**d**	Injection	171,502	26.4	46,710	21.3	124,792	29
		**e**	Other	4,206	0.6	1,623	0.7	2,583	0.6
**Route of administration (secondary)**	**ROUTE2**	**a**	Oral	189,859	29.2	61,755	28.1	128,104	29.8
		**b**	Smoking	283,601	43.7	103,676	47.2	179,925	41.9
		**c**	Inhalation	85,434	13.2	26,993	12.3	58,441	13.6
		**d**	Injection	85,580	13.2	25,194	11.5	60,386	14
		**e**	Other	5,005	0.8	2,040	0.9	2,965	0.7
**Frequency of use at admission (primary)**	**FREQ1**	**a**	No use in past month	201,785	31.1	61,316	27.9	140,469	32.7
		**b**	Some use	186,790	28.8	61,046	27.8	125,744	29.3
		**c**	Daily use	260,904	40.2	97,296	44.3	163,608	38.1
**Frequency of use at discharge (primary)**	**FREQ1_D**	**a**	No use in past month	352,751	54.3	158,657	72.2	194,094	45.2
		**b**	Some use	166,892	25.7	32,064	14.6	134,828	31.4
		**c**	Daily use	129,836	20	28,937	13.2	100,899	23.5
**Age at first use (primary)**	**FRSTUSE1**	**a**	11 years and under	33,397	5.1	12,547	5.7	20,850	4.9
		**b**	12–14 years	113,169	17.4	41,973	19.1	71,196	16.6
		**c**	15–17 years	154,708	23.8	56,628	25.8	98,080	22.8
		**d**	18–20 years	114,827	17.7	38,804	17.7	76,023	17.7
		**e**	21–24 years	83,225	12.8	26,164	11.9	57,061	13.3
		**f**	25–29 years	67,519	10.4	19,961	9.1	47,558	11.1
		**g**	30 years and over	82,634	12.7	23,581	10.7	59,053	13.7
**Substance use disorder type**	**ALCDRUG**	**a**	Alcohol only	15	0	5	0	10	0
		**b**	Other drugs only	327,693	50.5	88,537	40.3	239,156	55.6
		**c**	Alcohol and other drugs	321,771	49.5	131,116	59.7	190,655	44.4
**Co-occurring mental and substance use disorders**	**PSYPROB**	**1**	Yes	366,663	56.5	109,016	49.6	257,647	59.9
		**2**	No	282,816	43.5	110,642	50.4	172,174	40.1

Regarding the disparities reported in this paper, starting with race and ethnicity, white patients make up 70.1% of the full sample, Black or African American patients make up 17.6%, and the remainder of races identified make up 12.3% of the sample. Non-Hispanic patients make up 88.9% of the sample. For primary income source, in the full sample, 27.1% of discharges were associated with patients who had income from wages/salary, 44.3% did not have or did not report a primary income source, and the remainder received income from public assistance, retirement/pension or disability, or other sources. For co-occurrence of a mental health disorder, 56.5% of discharges were associated with patients with at least one co-occurring mental health disorder while 43.5% were associated with patients without a co-occurring mental health disorder.

Full missing data details are reported in the S1 Appendix, with largest amount of missing data (>20%) occurring within variables for DSM diagnosis (DSMCRIT), frequency of use at discharge (primary) (FREQ_D) and living arrangement at discharge (LIVARAG_D).

### Virtual twins: First stage results

The resulting feature importance from this first stage random forest were as follows, with the scaled importance in parenthesis, where 1 is the most important: type of service discharged from (1.00), frequency of use of primary substance at discharge (0.58), DSM diagnosis (0.49), length of stay (0.47), age (0.29), secondary substance used (0.23), primary substance used (0.22), referral source (18.2), frequency of use of primary substance at admission (0.16), and employment status at discharge (0.15).

### Virtual twins: Second stage results

For the second stage results, we report the decision trees developed using R (package: h2o) applied to the disparity in question. All left branches mean “yes” the branching condition was met. All right branches mean “no” the branching condition was not met. The decimal values represent the increased probability of completing substance use disorder treatment due to being in the subgroup identified by the branching conditions. When higher, these decimal values indicate greater likelihood of completing treatment. The hues represent lower (lighter) or higher (darker) probabilities of completing treatment. The percentage indicates percentage of the discharges in the sample represented by the specific node.

**Fig 2:**
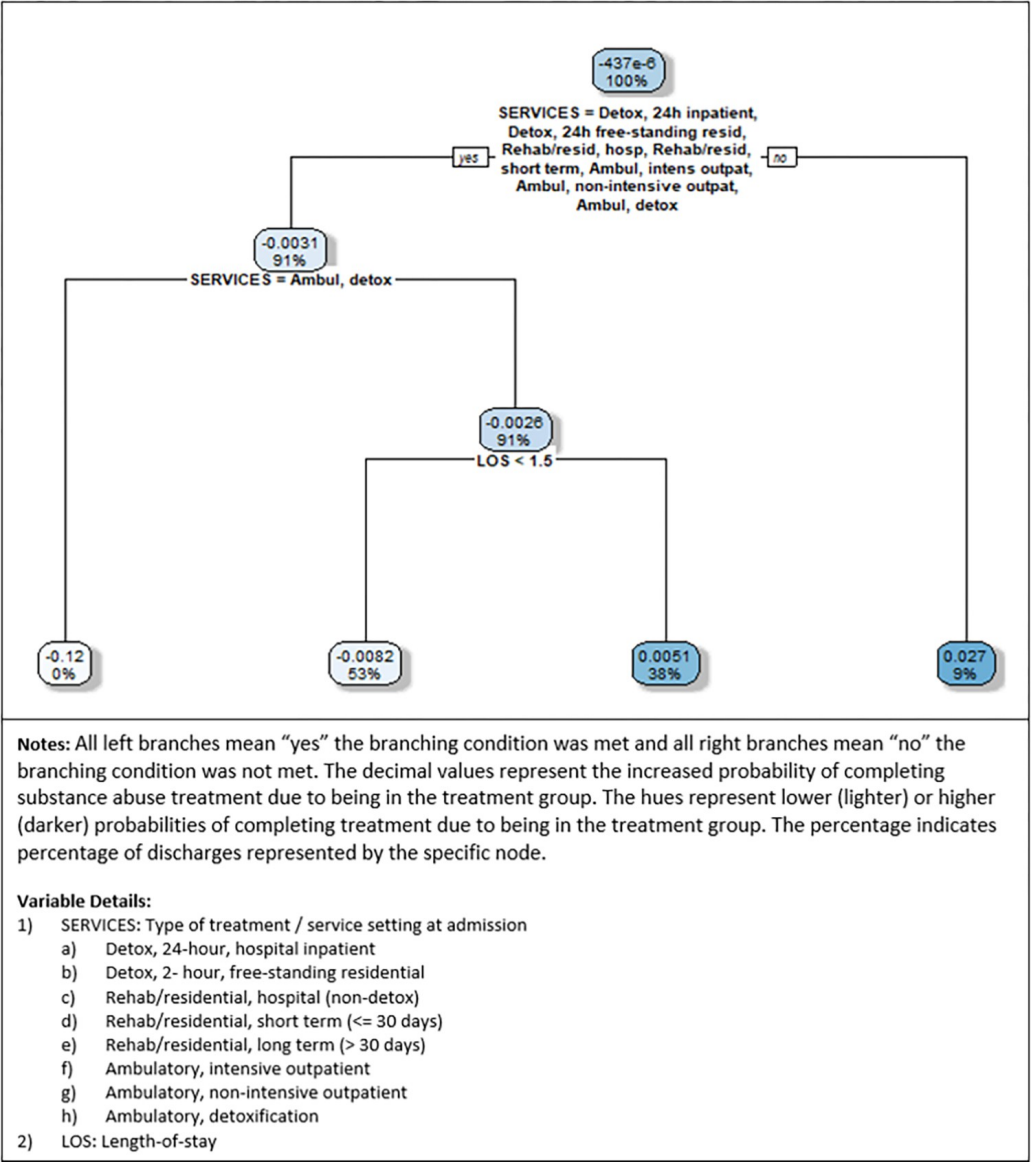
Race/ethnicity decision tree (P_1i_ = white non-Hispanic).

Fig 2 depicts the decision tree for race/ethnicity disparity, where the probability difference was calculated as P_1i_ (white non-Hispanic)–P_0i_ (all other races and ethnicities). Thus, the nodes represent the increased (or decreased) probability of completing treatment successfully when white non-Hispanic. Overall, the highest probability is 2.7% (representing 9% of the sample) when the service is rehab/residential, long term (>30 days), which is the only service not in the list of services specified in the branching node. This suggests that a racial disparity exists particularly for longer-term treatment. On the other end of the spectrum, we find that completing treatment successfully is 12% less likely for white non-Hispanic patients when admitted to ambulatory detox, but the percentage of the sample represented is near 0%, suggesting that this difference applies to few discharges. Disparities for other subgroups identified are less than 1%.

**Fig 3:**
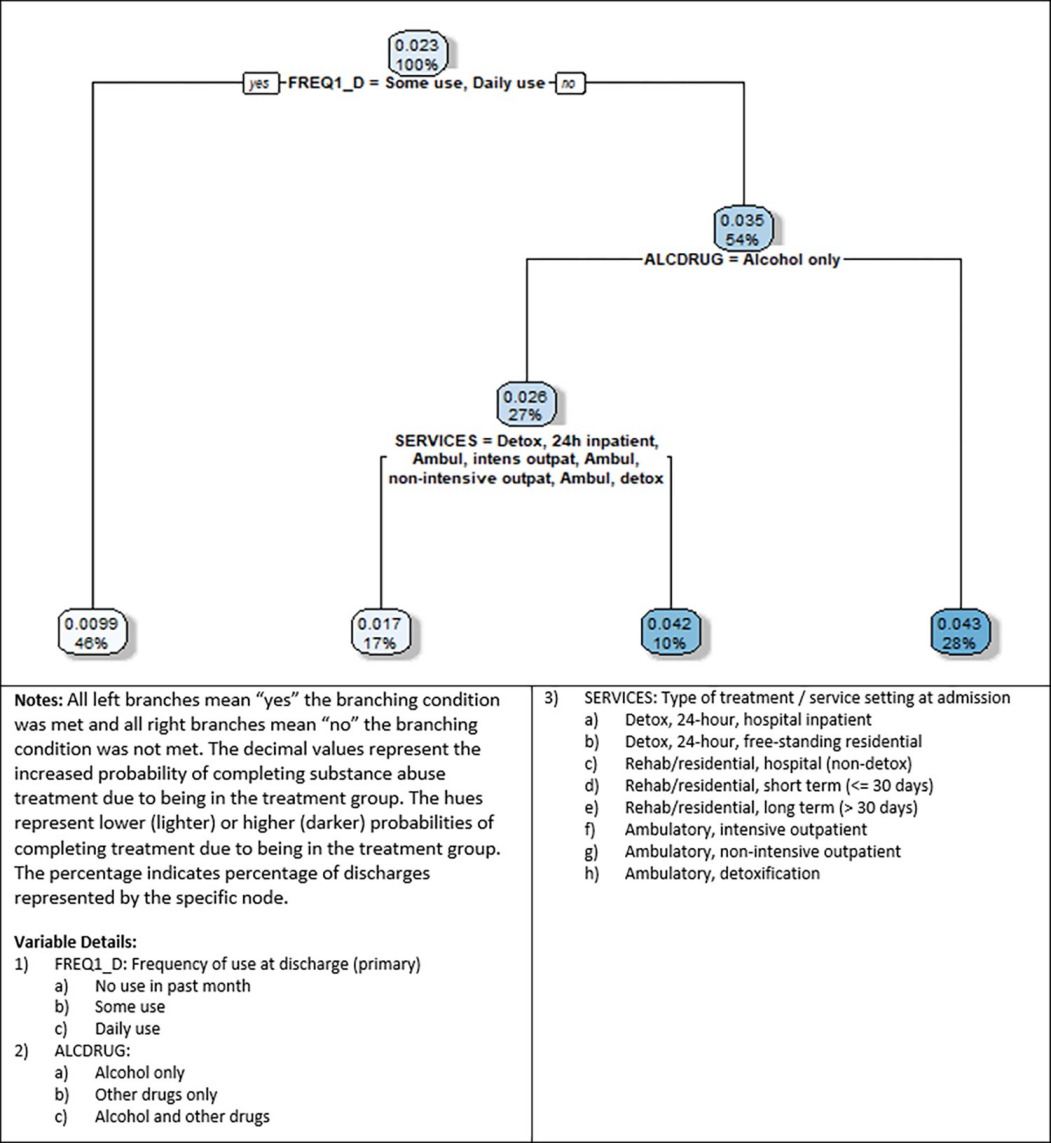
Income source decision tree (P_1i_ = wages/salary).

Fig 3 depicts the decision tree for income source disparity, where the probability difference was calculated as P_1i_ (wages/salary)–P_0i_ (all other income sources). The nodes represent the increased (or decreased) probability of completing treatment successfully when a regular source of job-related income is available. Overall, all the probabilities are positive, suggesting that those with job-related income are more likely to successfully complete treatment. Those who have not used in the past month (54% of the sample) have a 3.5% higher probability of completing treatment if their income source is from wages or salary. Further, one of the highest probabilities is 4.3%, representing 28% of the sample, for those with no use in the past month and using either drugs only or drugs in addition to alcohol use. The other highest probability is 4.2%, for those with no use in the past month, are only alcohol users, and who are either discharged from Detox 24-hour free-standing residential or any of the rehab/residential types of programs. We also note that these probabilities (4.3% and 4.2% respectively) are higher than the highest probability associated with racial disparities (2.7%), suggesting that income source disparities are somewhat higher than race/ethnicity disparities, for some subgroups.

**Fig 4:**
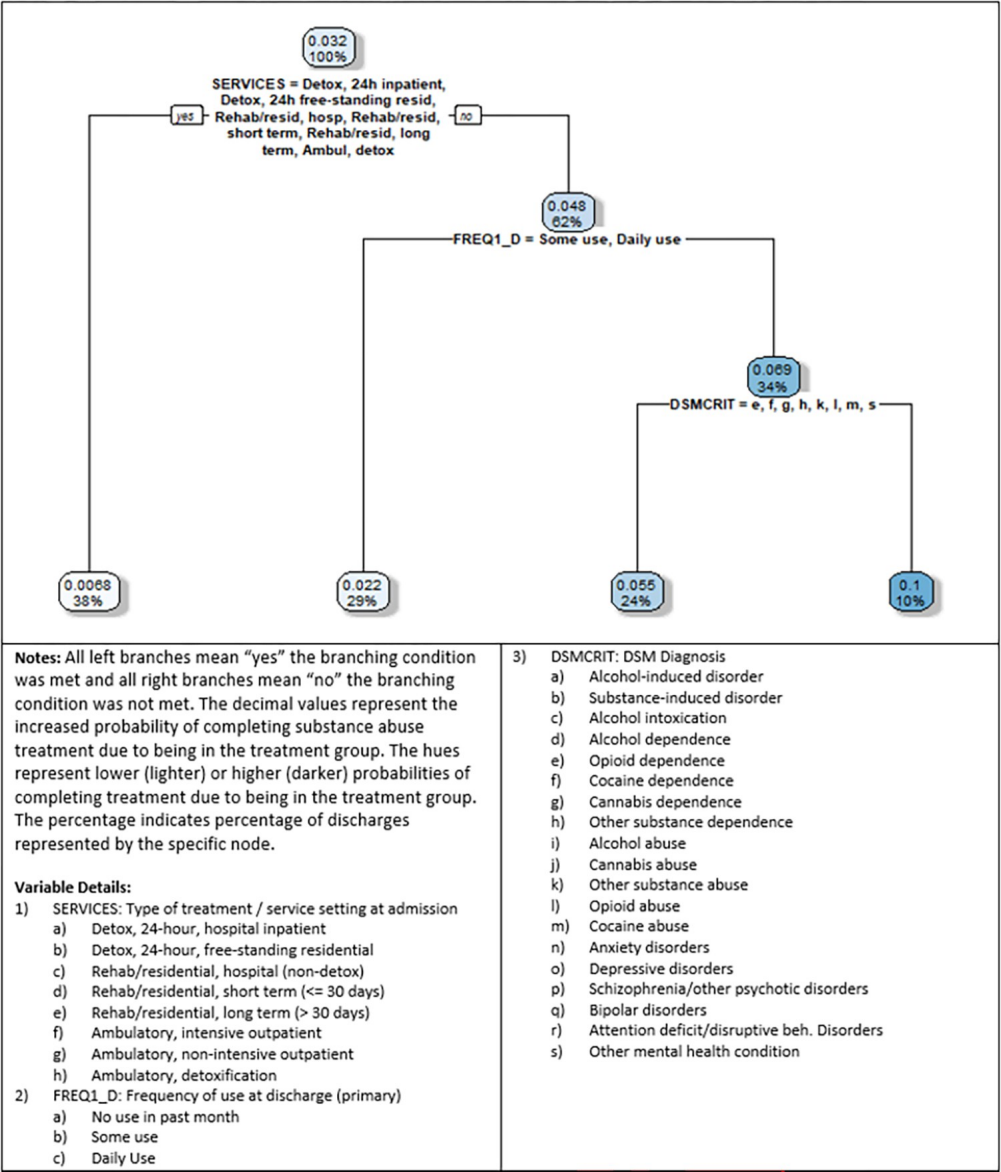
No co-occurring mental health disorder decision tree (P_1i_ = no co-occurring mental health disorder).

Fig 4 depicts the decision tree for no co-occurring mental health disorder disparity, where the probability difference was calculated as P_1i_ (no co-occurring substance use and mental health disorder)–P_0i_ (co-occurring). We note that this decision tree was grown for discharges where PSYPROB (co-occurring mental and substance use disorders) is equal to “No.” We mention this as the TEDS-D data also includes a variable called DSMCRIT (i.e., DSM diagnosis), that includes options for values for both substance use and mental health diagnoses, but each discharge is only assigned one primary diagnosis within this variable. Thus, it is impossible to tell with this variable if there is a co-occurring substance use and mental health diagnosis. The PSYPROB variable is a Yes/No variable that captures whether there are co-occurring substance and mental health diagnoses. While there is some overlap between PSYPROM and DSMCRIT, we based the tree on the PSYPROB variable, as it accurately reflects dual diagnoses.

The nodes in the tree represent the increased (or decreased) probability of completing treatment successfully when one does not have co-occurring substance use and mental health disorders. Overall, all the probabilities are positive, suggesting that one is more likely to successfully complete treatment if not also diagnosed with a mental health disorder. The highest probability of 10% (representing 10% of the sample) is for the subgroup of those discharged from ambulatory, outpatient services (either intensive or non-intensive), have not used in the past month, and with primary a DSMCRIT diagnosis of a (alcohol-induced disorder), b (substance-induced disorder), c (alcohol intoxication), d (alcohol dependence), i (alcohol abuse), j (cannabis abuse), n (anxiety disorders), o (depressive disorders), p (schizophrenia/other psychotic disorders), q (bipolar disorders), or r (attention deficit/disruptive behavior Disorders). We note that this probability (10.0%) is the highest observed in this study (when >1% of the sample is represented). We also note that even higher up in the tree, for the node with a 4.8% probability representing 62% of the sample (which is for those discharged from an ambulatory outpatient treatment program) is also higher than the probabilities observed in the other decision tree results (race and income from wages/job), when >1% of the sample is represented. These results are consistent with the results from the robustness checks (Fig 3 in S1 Appendix) Thus, we conclude that those with no mental health co-occurrence have the highest probability of completing treatment successfully.

### Robustness

First, we evaluated the potential impacts of imbalanced data, associated with our first stage dependent variable, by assessing accuracy as well as AUC, precision, recall and F1 scores for the balanced data using the Synthetic Minority Over-sampling Technique (SMOTE) (see the statistics in the S1 Appendix). We find that the out-of-sample statistics using SMOTE are very similar to the statistics resulting for the analyses run using the original data. Given that both precision and recall are high and consistent with each other, we conclude that the results of the prediction models are not imbalanced in favor of only one class (or a minority of classes).

Second, a potential issue with our first stage dependent variable (treatment completion) is that some of the unsuccessful completion categories, such as transfer, incarcerated, death, or other, may not reflect a disparity in treatment completion, but rather changes or issues that occurred outside of the control of the individual or treatment program. Some studies have addressed this issue by only focusing on planned discharges [15, 24] or by dropping detox related readmissions [15]. Thus, for robustness, we re-ran the analyses with a subset of the data for only two categories: treatment completed (coded as 1), and both dropped out of treatment and terminated by facility coded as 0, with all other observations for other reasons dropped. As can be seen in the S1 Appendix, while there are some minor differences in the results for these robustness checks, the probabilities and subgroups identified are very similar to the main analyses. We do note two differences, however. In the race/ethnicity disparity robustness check, for nodes representing >1% of the sample, one of the subgroups has a -1.1% probability (representing 49% of the sample) of successfully completing treatment. This suggests that disparities may be present in the other direction (i.e., in favor of minorities) in some cases. For the income source disparity robustness check, the highest disparity is 3.4% (representing 55% of the sample), which is a full percentage point lower than the highest reported income source disparity in the main results and is similar to the highest race/ethnicity probability percentage in its respective robustness check results (3.3%; representing 5% of the sample). Although these highest probabilities for income source and race/ethnicity disparity are similar, given that the robustness check for income source disparity represents a much higher percentage of the sample (55% vs. 5%), we maintain that disparities evaluated in this study, for certain subgroups, occur in this order: no mental health co-occurrence, income source (job related wages), racial/ethnicity (white non-Hispanic).

## Discussion

Leveraging a national dataset of substance use disorder treatment discharges for 2017–2019 in the U.S., this study has examined disparities in substance use disorder treatment completion. After evaluating several potential disparities, the three most prominent disparities found are: no co-occurrence of substance use and mental health disorders, income source, and race/ethnicity. Through application of a virtual twins method, which is a counterfactual approach used to identify subgroups subject to differences in outcomes, we find that disparities are indeed present and should be considered in more depth by researchers and practitioners alike.

Our primary finding is that, for the disparities considered in this study, the highest probability for successfully completing treatment when the subgroup represents more than 1% of the sample, is for one of the subgroups within the no mental health condition co-occurrence (10% more likely to complete treatment; representing 10% of the sample). The second highest is for a subgroup within the income source from wages/job decision tree (4.3% more likely to complete treatment; representing 28% of the sample). The third highest is for a white non-Hispanic subgroup in the race/ethnicity decision tree (2.7% more likely to complete treatment; representing 9% of the sample).

Prior studies have shown that racial disparities are present in substance use disorder treatment completion [14, 15]. Our findings confirm that racial/ethnic disparities persist, particularly when admitted to residential, long-term (>30 days) treatment programs. This finding suggests that disparities may exist when decisions are made as to which type of program to admit a patient to or retain within. This implies that biases associated with race or ethnicity should be particularly examined in the process of determining which program to refer or admit patients into as well as in treatment continuation decisions.

We also find that other disparities exist, that also require practitioner and policy maker attention. Subgroups associated with having job related income or not having a co-occurring mental health condition have the largest probabilities of successfully completing treatment completion, in this study. Prior work has shown that disparities are present for those with mental health conditions [36] and that co-occurrence of substance use disorder and mental health conditions is often associated with barriers to sufficient care [37]. Prior work has also shown that racial/ethnic minorities with lower income often lack equitable access to substance use disorder treatment [38]. However, to our knowledge, the heterogenous treatment effects associated with income source disparities and co-occurrence of substance use disorder and mental health disorders have not yet been fully considered in relation to substance use disorder treatment completion. Thus, we contribute by identifying additional subgroups for whom treatment completion is more or less likely.

Regarding mental health disorder co-occurrence, the highest treatment completion probabilities for this subgroup were for who were discharged from ambulatory (non-detox) services. Specifically, this suggests that more investments are likely needed in services for patients with dual-diagnoses and, if dual diagnosis patients are routed to ambulatory services, specialized programs or tailored resources may be needed to reduce this disparity.

Regarding income source, those with job-related income and who had not used their primary substance in the last 30 days upon discharge were the most likely to complete treatment. This suggests that job retention or placement programs, for individuals who are willing and able to work, may reduce disparities in completion treatment. This may require that substance treatment also include either social programs or readily available connections to those offering such programs. Further, as is the case throughout health care, more emphasis on coordination between achieving treatment goals as well as achieving social goals may be required by those assisting patients in treatment.

This study is primarily limited by two data issues: missing data and data not submitted by some U.S. states (e.g., Georgia, Oregon, Washington, and West Virginia did not submit data to SAMHSA in some years). We sought to address these issues by analyzing available data across the entire U.S. (i.e., not just for specific states). Secondarily, this study is limited by not being able to observe effects for immutable characteristics for the same discharge (e.g., being white non-Hispanic and another race or ethnicity at the same time). The virtual twins analysis counterfactual design was specifically selected to address this issue.

Overall, this study has shown that disparities exist and persist in substance use disorder treatment completion. Given that this study is based on a national sample, substance use disorder treatment programs can use these results apply customized approaches toward mitigating disparity risk. For instance, while race/ethnicity is an important disparity to continue to consider, we also find that other types of disparities are present, suggesting that policy makers and practitioners consider at least income and co-occurring diagnoses, in addition to race and ethnicity, when making resource allocation and programmatic design decisions.

## Supporting information

S1 Appendix Supplementary tables and figures. (DOCX)
