# Supplementary material for: Use of machine learning to examine disparities in completion of substance use disorder treatment
Source: PLoS One. 2022 Sep 23;17(9):e0275054. doi: 10.1371/journal.pone.0275054 (PMC9506659; doi:10.1371/journal.pone.0275054)
Supplement: S1 Appendix — (DOCX) [file pone.0275054.s001.docx]

**Use of Machine Learning to Examine Disparities in Completion of Substance Use Disorder Treatment**

**SUPPLEMENTARY APPENDIX**

Sup Appendix Table 1 describes the out-of-sample prediction statistics using balanced data (SMOTE) with 50% down-sampling so that the samples have a 1:1 ratio between treatment and control

| **Sup Appendix Table 1: Out-of-sample prediction accuracy** | | | | | | **Sup Appendix Table 1: Out-of-sample prediction statistics** |
| --- | --- | --- | --- | --- | --- | --- |
|  | **Random Forest** | **XGBoost** | **Logistic Reg.** | **Neural Network** | **Decision Tree** |  |
| **Accuracy** | 0.800 | 0.792 | 0.774 | 0.743 | 0.726 |  |
| **AUC** | 0.881 | 0.868 | 0.851 | 0.818 | 0.753 |  |
| **Precision** | 0.655 | 0.647 | 0.628 | 0.641 | 0.573 |  |
| **Recall** | 0.830 | 0.806 | 0.798 | 0.577 | 0.766 |  |
| **F1** | 0.732 | 0.718 | 0.703 | 0.425 | 0.656 |  |

Sup Appendix Table 2 describes missing data.

| **Sup Appendix Table 2: Missing data description for TEDS-D 2017-2019 combined data** | | | | |
| --- | --- | --- | --- | --- |
| **Variable Description** | **Var. Abbr.** | **# Missing** | **% Missing** |  |
| **DSM Diagnosis** | **DSMCRIT** | 1,499,754 | 29.7% |  |
| **Frequency of use at discharge (primary)** | **FREQ1_D** | 1,414,472 | 28.0% |  |
| **Living arrangement at discharge** | **LIVARAG_D** | 1,035,258 | 20.5% |  |
| **Employment status at discharge** | **EMPLOY_D** | 943,764 | 18.7% |  |
| **Arrests in past 30 days prior to discharge** | **ARRESTS_D_num** | 946,319 | 18.7% |  |
| **Co-occurring mental and substance use disorders** | **PSYPROB** | 684,041 | 13.5% |  |
| **Frequency of use at admission (primary)** | **FREQ1** | 550,785 | 10.9% |  |
| **Arrests in past 30 days prior to admission** | **ARRESTS_num** | 516,651 | 10.2% |  |
| **Previous substance use treatment episodes** | **NOPRIOR_num** | 428,236 | 8.5% |  |
| **Employment status at admission** | **EMPLOY** | 326,786 | 6.5% |  |
| **Referral Source** | **PSOURCE** | 238,795 | 4.7% |  |
| **Substance use at admission (secondary)** | **SUB2** | 175,889 | 3.5% |  |
| **Substance use at admission (primary)** | **SUB1** | 173,493 | 3.4% |  |
| **Ethnicity** | **ETHNIC** | 155,573 | 3.1% |  |
| **Race** | **RACE** | 138,662 | 2.7% |  |
| **Notes:** Only variables with missing data are included in this table. If a variable is not present here, it had 0% missing data. The table is sorted by missing data percentage, with the largest values appearing at the top. | | | | |

The following variables from TEDS-D were dropped from the analyses due to high collinearity or high missing values:

- Health insurance (HLTHINS)
- Payment source, primary (expected or actual) (PRIMPAY)
- Detailed not in labor force category at admission (DETNLF)
- Detailed not in labor force category at discharge (DETNLF_D)
- Route of administration (tertiary) (ROUTE3)
- Age at first use (secondary) (FRSTUSE2)
- Age at first use (tertiary) (FRSTUSE3)
- Pregnant at admission (PREG)
- Frequency of use at admission (secondary) (FREQ2)
- Frequency of use at discharge (tertiary) (FREQ3_D)
- Substance use at discharge (primary) (SUB1_D)
- Substance use at discharge (secondary) (SUB2_D)
- Substance use at discharge (tertiary) (SUB3_D)
- Type of treatment service/setting at discharge (SERVICES_D)
- Detailed criminal justice referral (DETCRIM)
- Frequency of use at discharge (secondary) (FREQ2_D)
- Substance use at admission (tertiary) (SUB3)
- Frequency of use at admission (tertiary) (FREQ3)
- Medication-assisted opioid therapy (METHUSE)
- Attendance at substance use self-help groups in past 30 days prior to admission (FREQ_ATND_SELF_HELP)
- Attendance at substance use self-help groups in past 30 days prior to discharge (FREQ_ATND_SELF_HELP_D)
- Alcohol reported at admission (ALCFLG)
- Heroin reported at admission (HERFLG)
- Other tranquilizers reported at admission (TRNQFLG)

Sup Appendix Table 3 describes the data both with and without rows that have at least one variable with missing data.

| **Sup Appendix Table 3: TEDS-D (2017-2019) descriptive statistics for all variables with and without rows with missing data** | | | | | | | |
| --- | --- | --- | --- | --- | --- | --- | --- |
|  |  |  | **Categorical Description** | **Without Missingness** | | **With Missingness** | |
| **Variable** | **Abbr.** | **Value** |  | **Freq** | **%** | **Freq** | **%** |
| **Year of client's discharge from treatment** | **DISYR** |  | 2017 | 162,063 | 25 | 1,499,144 | 34.1 |
|  |  |  | 2018 | 232,365 | 35.8 | 1,434,001 | 32.6 |
|  |  |  | 2019 | 255,051 | 39.3 | 1,467,452 | 33.3 |
| **Reason for discharge** | **REASON** | **a** | **Treatment completed** | 219,658 | 33.8 | 1,888,339 | 42.9 |
|  |  | **b** | Dropped out of treatment | 148,174 | 22.8 | 1,119,905 | 25.4 |
|  |  | **c** | Terminated by facility | 49,505 | 7.6 | 248,380 | 5.6 |
|  |  | **d** | Transferred to another treatment program | 189,410 | 29.2 | 879,711 | 20 |
|  |  | **e** | Incarcerated | 14,954 | 2.3 | 69,481 | 1.6 |
|  |  | **f** | Death | 1,156 | 0.2 | 9,879 | 0.2 |
|  |  | **g** | Other | 26,622 | 4.1 | 184,902 | 4.2 |
| **Race** | **RACE** | **a** | Alaskan Native | 4,457 | 0.7 | 6,988 | 0.2 |
|  |  | **b** | American Indian | 12,915 | 2 | 104,839 | 2.5 |
|  |  | **c** | Asian or Pacific Islander | 16 | 0 | 1,254 | 0 |
|  |  | **d** | Black or African American | 114,081 | 17.6 | 767,482 | 18 |
|  |  | **e** | White | 455,413 | 70.1 | 2,881,306 | 67.6 |
|  |  | **f** | Asian | 3,402 | 0.5 | 29,235 | 0.7 |
|  |  | **g** | Other single race | 44,029 | 6.8 | 348,105 | 8.2 |
|  |  | **h** | Two or more races | 8,730 | 1.3 | 102,923 | 2.4 |
|  |  | **i** | Native Hawaiian or Other Pacific Islander | 6,436 | 1 | 19,803 | 0.5 |
| **Ethnicity** | **ETHNIC** | **a** | Puerto Rican | 25,619 | 3.9 | 154,072 | 3.6 |
|  |  | **b** | Mexican | 13,979 | 2.2 | 197,763 | 4.7 |
|  |  | **c** | Cuban or other specific Hispanic | 19,484 | 3 | 134,816 | 3.2 |
|  |  | **d** | Not of Hispanic or Latino Origin | 577,254 | 88.9 | 3,644,517 | 85.9 |
|  |  | **e** | Hispanic or Latino, origin not specified | 13,143 | 2 | 113,856 | 2.7 |
| **Gender (Biological)** | **GENDER** | **a** | Male | 402,735 | 62 | 2,855,101 | 64.9 |
|  |  | **b** | Female | 246,744 | 38 | 1,543,830 | 35.1 |
| **Martial Status** | **MARSTAT** | **a** | Never married | 424,619 | 65.4 | 2,185,473 | 66 |
|  |  | **b** | Now married | 81,081 | 12.5 | 423,574 | 12.8 |
|  |  | **c** | Separated | 44,916 | 6.9 | 196,565 | 5.9 |
|  |  | **d** | Divorced/widowed | 98,863 | 15.2 | 504,789 | 15.2 |
| **Education** | **EDUC** | **a** | < Grade 9 | 34,945 | 5.4 | 243,553 | 6 |
|  |  | **b** | Grades 9 to 11 | 146,245 | 22.5 | 843,008 | 20.9 |
|  |  | **c** | Grade 12 (or GED) | 307,664 | 47.4 | 1,891,502 | 46.9 |
|  |  | **d** | 1-3 years of post-secondary | 127,879 | 19.7 | 803,692 | 19.9 |
|  |  | **e** | 4+ years of post-secondary | 32,746 | 5 | 248,214 | 6.2 |
| **Veteran Status** | **VET** | **a** | Yes | 17,100 | 2.6 | 109,386 | 2.8 |
|  |  | **b** | No | 632,379 | 97.4 | 3,772,424 | 97.2 |
| **Sources of income/support** | **PRIMINC** | **a** | Wages/salary | 166,161 | 25.6 | 706,069 | 29.8 |
|  |  | **b** | Public assistance | 53,294 | 8.2 | 184,307 | 7.8 |
|  |  | **c** | Retirement/pension, disability | 38,081 | 5.9 | 168,238 | 7.1 |
|  |  | **d** | Other | 117,726 | 18.1 | 408,208 | 17.2 |
|  |  | **e** | None | 274,217 | 42.2 | 906,212 | 38.2 |
| **Age at admission (binned)** | **AGE** | **a** | 12-14 years | 2,397 | 0.4 | 24,212 | 0.6 |
|  |  | **b** | 15-17 years | 14,019 | 2.2 | 115,405 | 2.6 |
|  |  | **c** | 18-20 years | 20,224 | 3.1 | 133,983 | 3 |
|  |  | **d** | 21-24 years | 61,586 | 9.5 | 370,881 | 8.4 |
|  |  | **e** | 25-29 years | 125,202 | 19.3 | 759,699 | 17.3 |
|  |  | **f** | 30-34 years | 119,503 | 18.4 | 740,828 | 16.8 |
|  |  | **g** | 35-39 years | 96,632 | 14.9 | 609,545 | 13.9 |
|  |  | **h** | 40-44 years | 61,928 | 9.5 | 426,063 | 9.7 |
|  |  | **i** | 45-49 years | 51,372 | 7.9 | 393,863 | 9 |
|  |  | **j** | 50-54 years | 47,235 | 7.3 | 364,831 | 8.3 |
|  |  | **k** | 55-64 years | 45,188 | 7 | 403,342 | 9.2 |
|  |  | **l** | 65 years and older | 4,193 | 0.6 | 57,945 | 1.3 |
| **Employment status at admission** | **EMPLOY** | **a** | Full-time | 106,412 | 16.4 | 714,025 | 17.5 |
|  |  | **b** | Part-time | 43,414 | 6.7 | 299,696 | 7.4 |
|  |  | **c** | Unemployed | 251,461 | 38.7 | 1,631,828 | 40.1 |
|  |  | **d** | Not in labor force | 248,192 | 38.2 | 1,428,262 | 35.1 |
| **Employment status at discharge** | **EMPLOY_D** | **a** | Full-time | 125,296 | 19.3 | 724,394 | 21 |
|  |  | **b** | Part-time | 48,646 | 7.5 | 283,306 | 8.2 |
|  |  | **c** | Unemployed | 243,200 | 37.4 | 1,365,174 | 39.5 |
|  |  | **d** | Not in labor force | 232,337 | 35.8 | 1,083,959 | 31.4 |
| **Living arrangement at admission** | **LIVARAG_A** | **a** | Homeless | 107,181 | 16.5 | 648,271 | 16.3 |
|  |  | **b** | Dependent living | 119,742 | 18.4 | 722,020 | 18.1 |
|  |  | **c** | Independent living | 422,556 | 65.1 | 2,618,674 | 65.6 |
| **Living arrangement at discharge** | **LIVARAG_D** | **a** | Homeless | 89,380 | 13.8 | 415,734 | 12.4 |
|  |  | **b** | Dependent living | 135,898 | 20.9 | 755,599 | 22.5 |
|  |  | **c** | Independent living | 424,201 | 65.3 | 2,194,006 | 65.2 |
| **Arrests in past 30 days prior to admission** | **ARRESTS** | **a** | None | 595,125 | 91.6 | 3,576,526 | 92.1 |
|  |  | **b** | Once | 47,759 | 7.4 | 255,352 | 6.6 |
|  |  | **c** | Two or more times | 6,595 | 1 | 52,068 | 1.3 |
| **Arrests in past 30 days prior to discharge** | **ARRESTS_D** | **a** | None | 601,854 | 92.7 | 3,256,638 | 94.3 |
|  |  | **b** | Once | 37,010 | 5.7 | 153,537 | 4.4 |
|  |  | **c** | Two or more times | 10,615 | 1.6 | 44,103 | 1.3 |
| **Previous substance use treatment episodes** | **NOPRIOR** | **a** | No prior treatment episodes | 200,009 | 30.8 | 1,591,654 | 40.1 |
|  |  | **b** | One or more prior treatment episodes | 449,470 | 69.2 | 2,380,707 | 59.9 |
| **Type of treatment/ service setting at admission** | **SERVICES** | **a** | Detox, 24 hour, hospital inpatient | 6,897 | 1.1 | 124,575 | 2.8 |
|  |  | **b** | Detox, 24 hour, free-standing residential | 48,287 | 7.4 | 718,901 | 16.3 |
|  |  | **c** | Rehab/residential, hospital (non-detox) | 346 | 0.1 | 12,486 | 0.3 |
|  |  | **d** | Rehab/residential, short term (<= 30 days) | 130,461 | 20.1 | 400,384 | 9.1 |
|  |  | **e** | Rehab/residential, long term (> 30 days) | 56,828 | 8.7 | 329,954 | 7.5 |
|  |  | **f** | Ambulatory, intensive outpatient | 98,844 | 15.2 | 591,277 | 13.4 |
|  |  | **g** | Ambulatory, non-intensive outpatient | 305,483 | 47 | 2,185,172 | 49.7 |
|  |  | **h** | Ambulatory, detoxification | 2,333 | 0.4 | 37,848 | 0.9 |
| **Length of stay in treatment (binned days)** | **LOS** | **a** | between 1 and 30 days | 370,434 | 57 | 2,311,573 | 52.5 |
|  |  | **b** | between 31 and 45 days | 44,270 | 6.8 | 292,027 | 6.6 |
|  |  | **c** | between 46 and 60 days | 32,631 | 5 | 225,125 | 5.1 |
|  |  | **d** | between 61 and 90 days | 51,719 | 8 | 374,591 | 8.5 |
|  |  | **e** | between 91 and 120 days | 38,404 | 5.9 | 294,542 | 6.7 |
|  |  | **f** | between 121 and 180 days | 43,901 | 6.8 | 326,169 | 7.4 |
|  |  | **g** | between 181 and 365 days | 49,703 | 7.7 | 347,393 | 7.9 |
|  |  | **h** | greater than 365 days | 18,417 | 2.8 | 229,177 | 5.2 |
| **Referral Source** | **PSOURCE** | **a** | Individual (includes self-referral) | 227,636 | 35 | 1,893,786 | 45.5 |
|  |  | **b** | Alcohol/drug use care provider | 70,792 | 10.9 | 385,524 | 9.3 |
|  |  | **c** | Other health care provider | 43,999 | 6.8 | 323,818 | 7.8 |
|  |  | **d** | School (educational) | 1,865 | 0.3 | 24,208 | 0.6 |
|  |  | **e** | Employer/EAP | 2,981 | 0.5 | 15,076 | 0.4 |
|  |  | **f** | Other community referral | 91,898 | 14.1 | 425,597 | 10.2 |
|  |  | **g** | Court/criminal justice referral/DUI/DWI | 210,308 | 32.4 | 1,093,793 | 26.3 |
| **DSM diagnosis (SuDS 4 or SuCDS 19)** | **DSMCRIT** | **a** | Alcohol-induced disorder | 2,068 | 0.3 | 29,043 | 1 |
|  |  | **b** | Substance-induced disorder | 16,332 | 2.5 | 86,229 | 3 |
|  |  | **c** | Alcohol intoxication | 19,504 | 3 | 71,990 | 2.5 |
|  |  | **d** | Alcohol dependence | 103,287 | 15.9 | 665,580 | 22.9 |
|  |  | **e** | Opioid dependence | 176,549 | 27.2 | 869,192 | 30 |
|  |  | **f** | Cocaine dependence | 28,756 | 4.4 | 124,662 | 4.3 |
|  |  | **g** | Cannabis dependence | 44,891 | 6.9 | 197,470 | 6.8 |
|  |  | **h** | Other substance dependence | 96,169 | 14.8 | 265,308 | 9.1 |
|  |  | **i** | Alcohol abuse | 15,605 | 2.4 | 126,278 | 4.4 |
|  |  | **j** | Cannabis abuse | 12,688 | 2 | 103,754 | 3.6 |
|  |  | **k** | Other substance abuse | 10,242 | 1.6 | 48,915 | 1.7 |
|  |  | **l** | Opioid abuse | 7,772 | 1.2 | 38,298 | 1.3 |
|  |  | **m** | Cocaine abuse | 3,396 | 0.5 | 19,151 | 0.7 |
|  |  | **n** | Anxiety disorders | 5,083 | 0.8 | 6,169 | 0.2 |
|  |  | **o** | Depressive disorders | 6,861 | 1.1 | 10,541 | 0.4 |
|  |  | **p** | Schizophrenia/other psychotic disorders | 1,923 | 0.3 | 2,541 | 0.1 |
|  |  | **q** | Bipolar disorders | 2,824 | 0.4 | 4,692 | 0.2 |
|  |  | **r** | Attention deficit/disruptive beh. disorders | 366 | 0.1 | 938 | 0 |
|  |  | **s** | Other mental health condition | 95,163 | 14.7 | 230,092 | 7.9 |
| **Substance use at admission (primary)** | **SUB1** | **a** | None | - | 0 | 98,368 | 2.3 |
|  |  | **b** | Alcohol | 168,218 | 25.9 | 1,378,931 | 32.6 |
|  |  | **c** | Cocaine/crack | 49,874 | 7.7 | 234,535 | 5.5 |
|  |  | **d** | Marijuana/hashish | 76,767 | 11.8 | 526,337 | 12.5 |
|  |  | **e** | Heroin | 173,920 | 26.8 | 1,031,406 | 24.4 |
|  |  | **f** | Non-prescription methadone | 1,661 | 0.3 | 8,889 | 0.2 |
|  |  | **g** | Other opiates and synthetics | 56,429 | 8.7 | 323,127 | 7.6 |
|  |  | **h** | PCP | 2,260 | 0.3 | 10,692 | 0.3 |
|  |  | **i** | Hallucinogens | 1,182 | 0.2 | 4,625 | 0.1 |
|  |  | **j** | Methamphetamines/speed | 96,848 | 14.9 | 495,547 | 11.7 |
|  |  | **k** | Other amphetamines | 6,070 | 0.9 | 21,661 | 0.5 |
|  |  | **l** | Other stimulants | 982 | 0.2 | 5,408 | 0.1 |
|  |  | **m** | Benzodiazepines | 9,314 | 1.4 | 45,642 | 1.1 |
|  |  | **n** | Other tranquilizers | 81 | 0 | 411 | 0 |
|  |  | **o** | Barbiturates | 151 | 0 | 2,752 | 0.1 |
|  |  | **p** | Other sedatives or hypnotics | 714 | 0.1 | 5,465 | 0.1 |
|  |  | **q** | Inhalants | 386 | 0.1 | 1,956 | 0 |
|  |  | **r** | Over-the-counter medications | 317 | 0 | 1,506 | 0 |
|  |  | **s** | Other drugs | 4,305 | 0.7 | 29,846 | 0.7 |
| **Substance use at admission (secondary)** | **SUB2** | **a** | None | 3,060 | 0.5 | 2,143,222 | 50.7 |
|  |  | **b** | Alcohol | 104,041 | 16 | 369,984 | 8.8 |
|  |  | **c** | Cocaine/crack | 117,246 | 18.1 | 427,576 | 10.1 |
|  |  | **d** | Marijuana/hashish | 169,697 | 26.1 | 530,234 | 12.6 |
|  |  | **e** | Heroin | 40,725 | 6.3 | 139,987 | 3.3 |
|  |  | **f** | Non-prescription methadone | 2,421 | 0.4 | 6,708 | 0.2 |
|  |  | **g** | Other opiates and synthetics | 52,536 | 8.1 | 150,304 | 3.6 |
|  |  | **h** | PCP | 1,967 | 0.3 | 6,291 | 0.1 |
|  |  | **i** | Hallucinogens | 2,469 | 0.4 | 6,965 | 0.2 |
|  |  | **j** | Methamphetamines/speed | 73,852 | 11.4 | 233,231 | 5.5 |
|  |  | **k** | Other amphetamines | 7,387 | 1.1 | 18,639 | 0.4 |
|  |  | **l** | Other stimulants | 3,453 | 0.5 | 9,167 | 0.2 |
|  |  | **m** | Benzodiazepines | 35,957 | 5.5 | 124,894 | 3 |
|  |  | **n** | Other tranquilizers | 170 | 0 | 783 | 0 |
|  |  | **o** | Barbiturates | 504 | 0.1 | 1,471 | 0 |
|  |  | **p** | Other sedatives or hypnotics | 2,051 | 0.3 | 9,572 | 0.2 |
|  |  | **q** | Inhalants | 643 | 0.1 | 1,470 | 0 |
|  |  | **r** | Over-the-counter medications | 549 | 0.1 | 2,117 | 0.1 |
|  |  | **s** | Other drugs | 30,751 | 4.7 | 42,093 | 1 |
| **Route of administration (primary)** | **ROUTE1** | **a** | Oral | 218,128 | 33.6 | 1,679,489 | 41 |
|  |  | **b** | Smoking | 162,850 | 25.1 | 1,035,423 | 25.3 |
|  |  | **c** | Inhalation | 92,793 | 14.3 | 466,300 | 11.4 |
|  |  | **d** | Injection | 171,502 | 26.4 | 882,116 | 21.5 |
|  |  | **e** | Other | 4,206 | 0.6 | 34,471 | 0.8 |
| **Route of administration (secondary)** | **ROUTE2** | **a** | Oral | 189,859 | 29.2 | 641,421 | 31 |
|  |  | **b** | Smoking | 283,601 | 43.7 | 898,557 | 43.4 |
|  |  | **c** | Inhalation | 85,434 | 13.2 | 262,100 | 12.7 |
|  |  | **d** | Injection | 85,580 | 13.2 | 254,057 | 12.3 |
|  |  | **e** | Other | 5,005 | 0.8 | 13,643 | 0.7 |
| **Frequency of use at admission (primary)** | **FREQ1** | **a** | No use in past month | 201,785 | 31.1 | 1,006,749 | 26.2 |
|  |  | **b** | Some use | 186,790 | 28.8 | 1,098,562 | 28.5 |
|  |  | **c** | Daily use | 260,904 | 40.2 | 1,744,501 | 45.3 |
| **Frequency of use at discharge (primary)** | **FREQ1_D** | **a** | No use in past month | 352,751 | 54.3 | 1,467,556 | 49.1 |
|  |  | **b** | Some use | 166,892 | 25.7 | 723,994 | 24.2 |
|  |  | **c** | Daily use | 129,836 | 20 | 794,575 | 26.6 |
| **Age at first use (primary)** | **FRSTUSE1** | **a** | 11 years and under | 33,397 | 5.1 | 236,795 | 5.8 |
|  |  | **b** | 12-14 years | 113,169 | 17.4 | 738,632 | 18.2 |
|  |  | **c** | 15-17 years | 154,708 | 23.8 | 1,022,305 | 25.1 |
|  |  | **d** | 18-20 years | 114,827 | 17.7 | 754,705 | 18.5 |
|  |  | **e** | 21-24 years | 83,225 | 12.8 | 493,442 | 12.1 |
|  |  | **f** | 25-29 years | 67,519 | 10.4 | 362,788 | 8.9 |
|  |  | **g** | 30 years and over | 82,634 | 12.7 | 460,184 | 11.3 |
|  |  |  | None | - | 0 | 271,861 | 6.2 |
| **Substance abuse type** | **ALCDRUG** | **a** | Alcohol only | 15 | 0 | 842,547 | 19.1 |
|  |  | **b** | Other drugs only | 327,693 | 50.5 | 2,245,099 | 51 |
|  |  | **c** | Alcohol and other drugs | 321,771 | 49.5 | 1,041,090 | 23.7 |
| **Co-occurring mental and substance use disorders** | **PSYPROB** | **1** | Yes | 366,663 | 56.5 | 1,493,865 | 40.2 |
|  |  | **2** | No | 282,816 | 43.5 | 2,222,691 | 59.8 |

**Robustness checks for disparities presented in the main paper**

For the robustness checks, treatment completed was coded as 1. Both dropped out of treatment and terminated by facility were coded as 0. All other observations for all the other reasons were dropped to account for potential differences from the main results if transfers or other reasons for being discharged occurred.

For all the figures below, all left branches mean “yes” the branching condition was met. All right branches mean “no” the branching condition was not met. The decimal values represent the increased probability of completing substance abuse treatment due to being in the subgroup identified by the branching conditions. When higher, these decimal values indicate greater likelihood of completing treatment. The hues represent lower (lighter) or higher (darker) probabilities of completing treatment. The percentage indicates percentage of the discharges in the sample represented by the specific node.

| 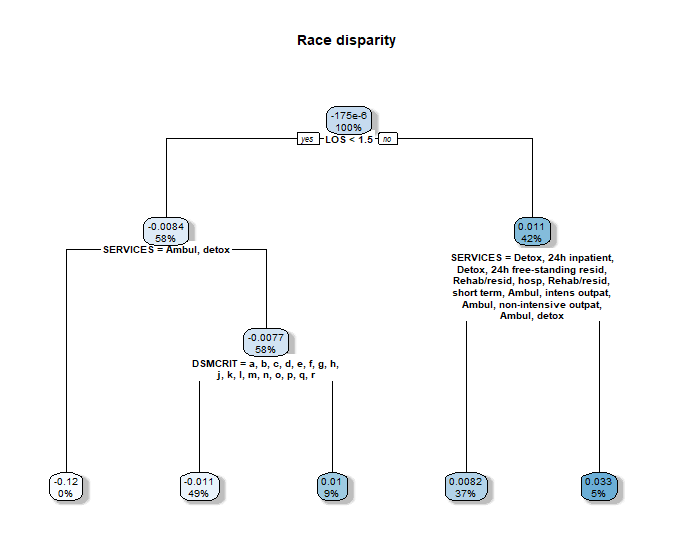 |
| --- |
| **Sup Appendix Figure 1:** Race/ethnicity disparity robustness (P_1i_ = white non-Hispanic) |

| 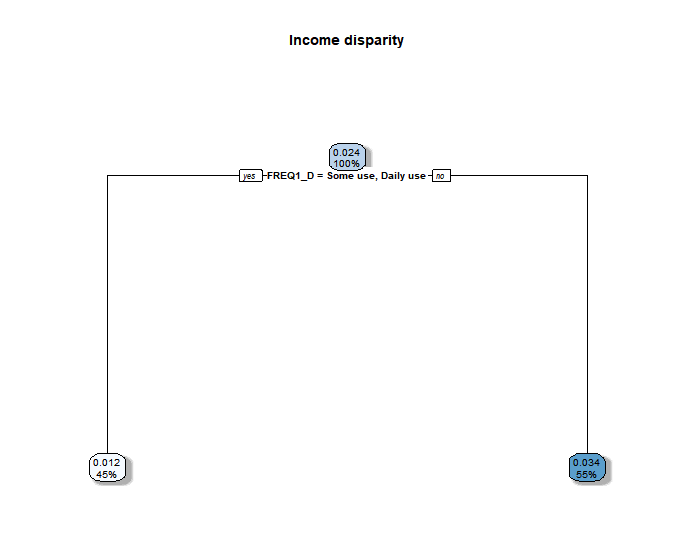 |
| --- |
| **Sup Appendix Figure 2:** Income source disparity robustness (P_1i_ = Wages/salary) |

| 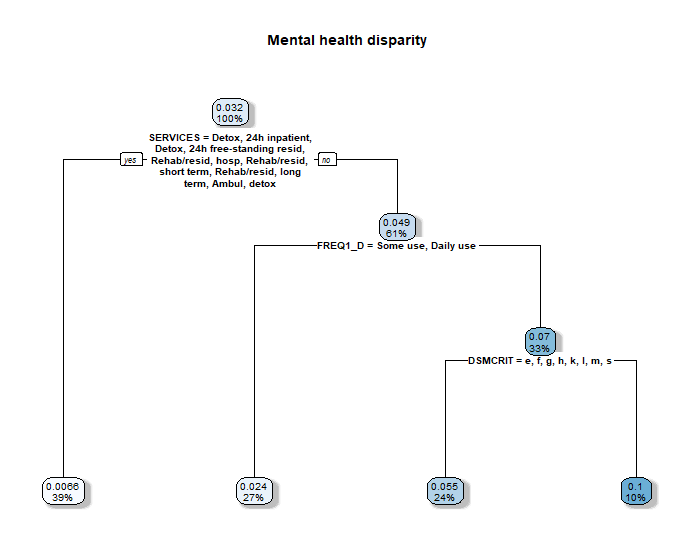 |
| --- |
| **Sup Appendix Figure 3:** No co-occurrence of mental health disorder disparity robustness (P_1i_ = No co-occurrence) |

**Additional Results**

The following figures depict the decision trees (and associated robustness checks) run for disparities evaluated but ultimately not presented in the main paper. We note that we also attempted to run decision trees for gender (biological) and health insurance, but these decision trees did not generate, suggesting that disparities could not be found for these variables. Thus, the results below are only for those disparities for which decisions trees could be generated.

| 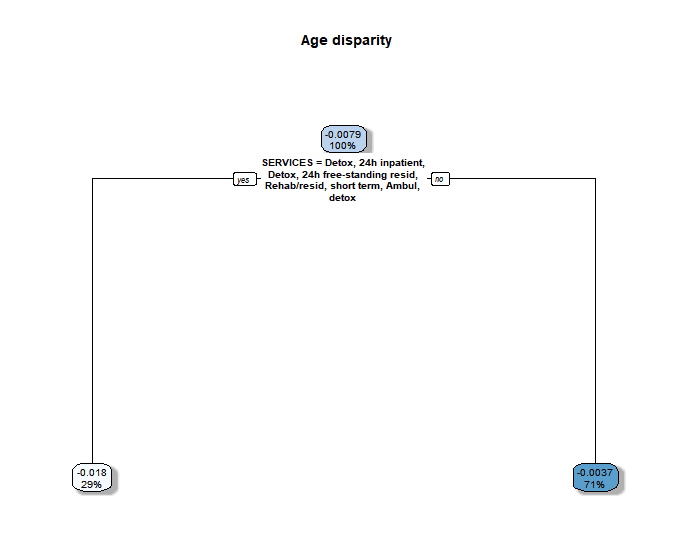 |
| --- |
| **Sup Appendix Figure 4:** Age disparity (P_1i_ = <35 years old) |

| 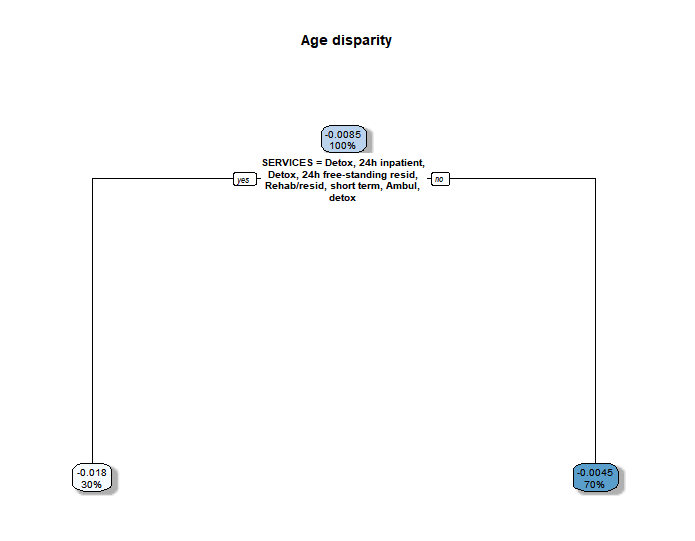 |
| --- |
| **Sup Appendix Figure 5:** Age disparity robustness (P_1i_ = <35 years old) |

| 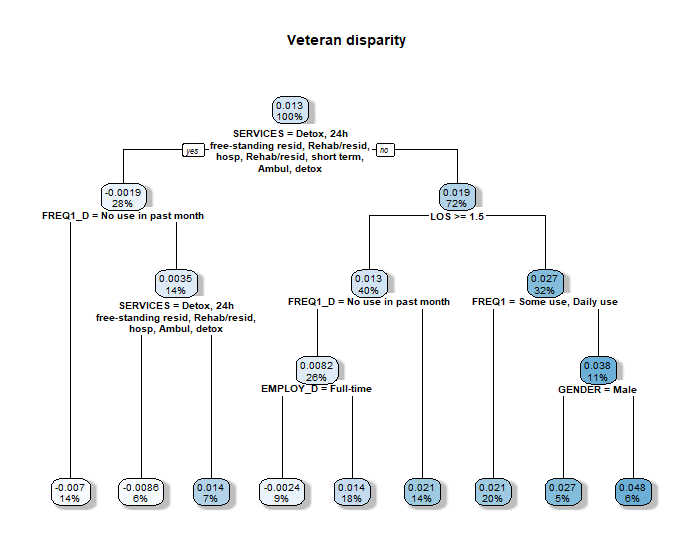 |
| --- |
| **Sup Appendix Figure 6:** Veteran status disparity (P_1i_ = Yes, veteran) |

(Note: No robustness is available for Veteran status disparity as the random forest did not generate when the robustness constraints were applied to the data. Given that robustness did not hold, we did not report these results in the main paper.)

| 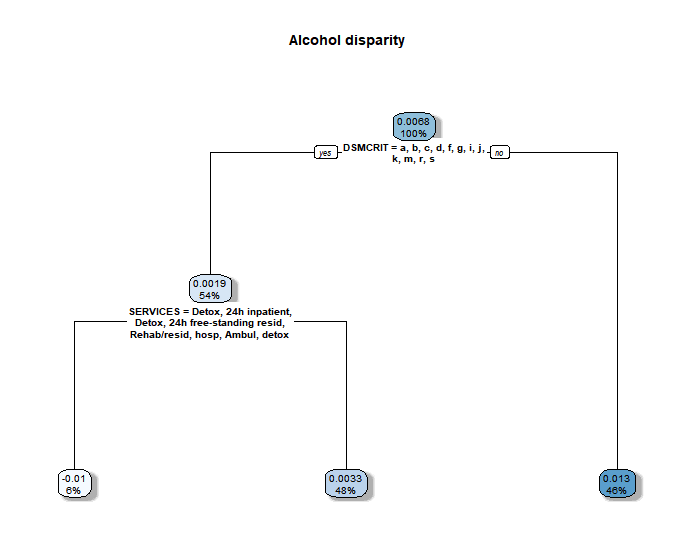 |
| --- |
| **Sup Appendix Figure 7:** Alcohol as primary substance disparity (P_1i_ = Alcohol as primary) |

| 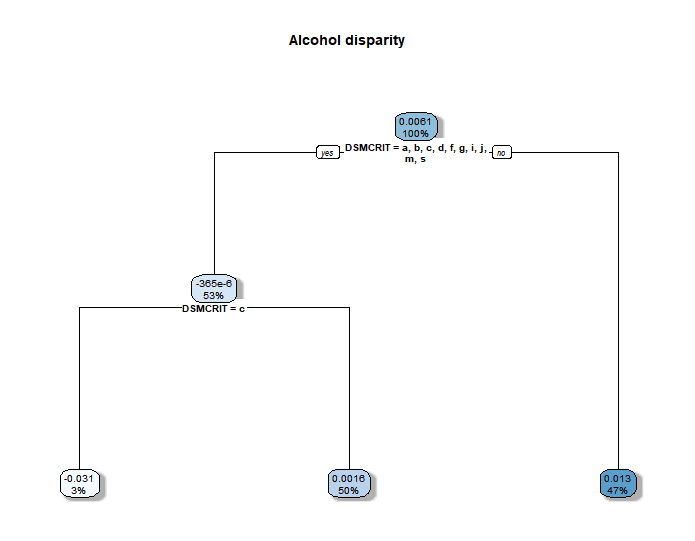 |
| --- |
| **Sup Appendix Figure 8:** Alcohol as primary substance disparity robustness (P_1i_ = Alcohol as primary) |

| 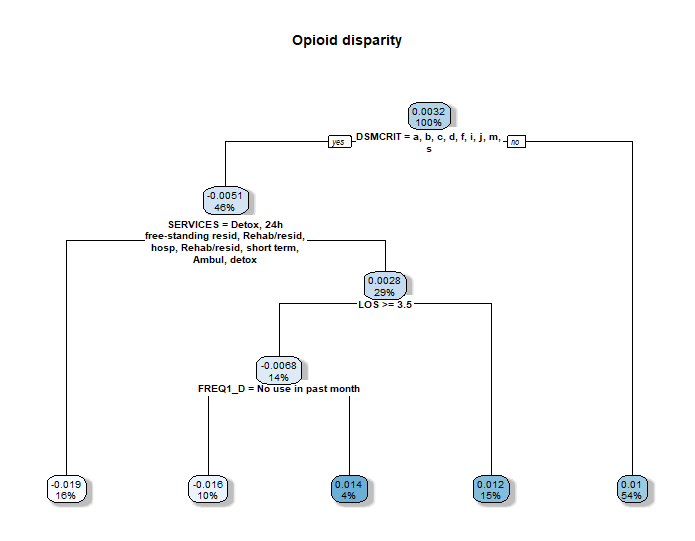 |
| --- |
| **Sup Appendix Figure 9:** Opioid as primary substance disparity (P_1i_ = Opioid as primary) |

| 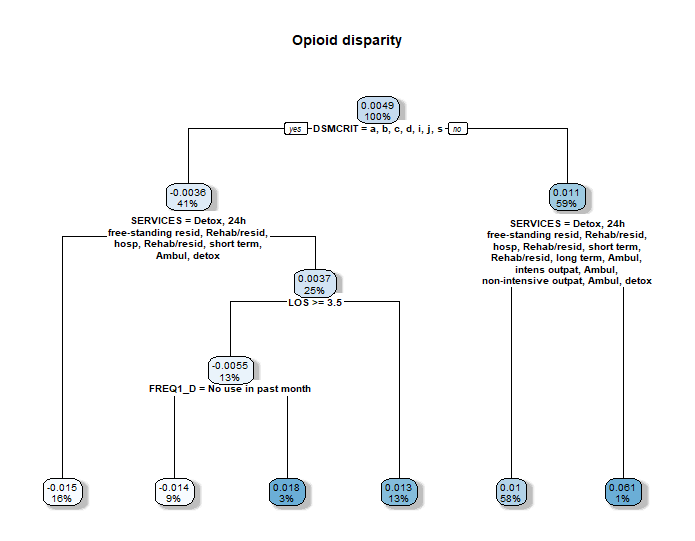 |
| --- |
| **Sup Appendix Figure 10:** Opioid as primary substance disparity robustness (P_1i_ = Opioid as primary) |
